# A Case of Extreme Gastroparesis due to an Occult Small Cell Cancer of the Lung

**DOI:** 10.1155/2013/182962

**Published:** 2013-12-04

**Authors:** J. A. C. M. Burger, B. Liberov, F. Yurd, R. J. L. F. Loffeld

**Affiliations:** ^1^Department of Internal Medicine, Zaans Medisch Centrum Zaandam, P.O. Box 210, 1500 EE Zaandam, The Netherlands; ^2^Department of Pulmonology, Zaans Medisch Centrum Zaandam, P.O. Box 210, 1500 EE Zaandam, The Netherlands

## Abstract

A patient with gastroparesis is presented. Ultimately the diagnosis of paraneoplastic gastroparesis due to an occult small cell cancer of the lung was made. The difficulties in the diagnostic process and the pathogenesis of this very rare manifestation are discussed.

## 1. Introduction

Gastroparesis, a chronic motility disorder, is characterized by upper gastrointestinal symptoms and leads to delayed gastric emptying. It negatively impacts morbidity due to inability of food intake, mortality due to the underlying cause, and discomfort that interferes with the quality of life [1]. There is no mechanical obstruction. Diabetes with poor glycaemic control and postsurgical causes represent the most common aetiologies. Gastroparesis also has been described as a complication of several malignancies, including gastric, pancreatic, gallbladder, oesophageal, and lung cancers, as well as leiomyosarcoma. The condition commonly manifests as upper gastrointestinal symptoms, including nausea, vomiting, postprandial fullness, early satiety, abdominal pain, and bloating [2]. Management of gastroparesis includes correction of malnutrition, dehydration and electrolyte imbalance, and relief of symptoms by use of prokinetic drugs.

Mostly the cause of gastroparesis is obvious, and the condition can be treated accordingly. The prevalence of paraneoplastic gastroparesis is unknown, and this entity probably is widely underrecognized and undertreated.

A case of extreme gastroparesis that ultimately proved to be due to an occult small cell carcinoma of the lung is presented.

## 2. Case Report

A 74-year-old male patient was presented in the emergency department of our hospital because of repeated vomiting. He was known with complaints of prostate enlargement and hypertension, smoked for many years, and used excessive amounts of alcohol. His medication comprised of hydrochlorothiazide, enalapril, omnic, tamsulosine, and omeprazole. Eight months earlier he was seen at the out-patient clinic because of malaise, pain in the proximal muscles, anorexia, a feeling of fullness in the abdomen, mild anaemia, and an erythrocyte sedimentation rate of 48 mm. Polymyalgia rheumatica was suspected and empiric treatment with steroids was started with a remarkable result. The complaints disappeared completely and the ESR normalized. The steroids were tapered and after six months definitely stopped. One month after stopping his complaints recurred. Despite this he went on a holiday to Spain. During this holiday, he was admitted because of abdominal pain, nausea, and vomiting. He underwent upper GI endoscopy, chest X-ray, and CT-scanning of the abdomen. The investigations revealed stomach retention and inflammation of the pyloric region. Abdominal CT-scan only showed diverticula in the sigmoid. After his return to the Netherlands he was admitted because of progressive vomiting.

On admission physical examination revealed no abnormalities, there were normal hemodynamics, and he was slightly dehydrated. Routine blood biochemistry was within the normal range, except for a slightly elevated serum calcium content which normalised after rehydration. Chest X-ray and a plain X-ray of the abdomen were normal. Revision of the CT-scan made in Spain did not give new findings. A new upper GI-endoscopy was done. Stomach retention was seen without clear anatomical obstruction. Because of the clinical suspicion of obstruction at the pyloric region, biopsy specimens were taken. These showed some signs of inflammation but no *H. pylori* or malignancy. Treatment with pro-kinetics (metoclopramide, domperidone, erythromycin, and pyridostigmine) was started without any effect. A nasojejunal tube was placed. During this placement repeat histological examination of random biopsies was done which ruled out eosinophilic enteritis. Because the patient did not tolerate the nasojejunal tube and because his clinical condition deteriorated parenteral feeding was started. A gastric emptying study with 13 MBq technetium nanocolloid was done. This showed extreme retention of the meal, not only in the stomach but also in the oesophagus. Five and a half hours after ingestion of the test meal the first sign of passage to the duodenum was observed.

Searching the literature and consulting an expert in the field of gastroparesis revealed that small cell cancer of the lung could be a possible cause. Revision of the chest X-rays did not show any sign of tumour nor enlarged lymph nodes. However, a CT-scan of the thorax showed enlarged mediastinal lymph nodes and a small lesion in the right lung. Additional bronchoscopy showed no endobronchial abnormalities. Endobronchial ultrasound with fine needle aspiration of a lymph node was consistent with small cell cancer of the lung. After the final diagnosis, anti-Hu antibodies were determined. These were highly positive (titre 25600, reference <400).

The final diagnosis was paraneoplastic gastroparesis as a result of an occult small cell cancer of the lung. The patient started with combination chemotherapy: etoposide and carboplatin. He tolerates this therapy well. A CT-scan after two courses of chemotherapy showed a remarkable reduction of the lymph nodes; also the magnitude of the stomach retention decreased significantly. However, repeated gastric emptying studies did not show any improvement. After completion of the chemotherapy the patient is in clinical remission but still needs parenteral feeding. Retrospectively, the small cell cancer of the lung with the beginning of gastroparesis already had to be present eight months earlier. The complaints responded well to treatment with steroids. This probably was due to an effect of steroids on the formation of anti-Hu antibodies by the tumour.

## 3. Discussion

Gastroparesis as the first symptom of an occult neoplasm easily evades the diagnostic thought especially when diagnostic workup has not detected any apparent cause. This case is a clear representation of the difficulties in the diagnostic process. At presentation the first clinical diagnosis was obstruction due to a gastric cancer located in the gastric outflow tract.

Generally, paraneoplastic syndromes occur in 7–15% of cancer patients and can involve all the organic systems of the human body with different frequencies [3]. Neurological involvement is found in only 0.01% of cancer patients and can be sensory, motor, mixed somatic, or autonomic leading to various syndromes and disorders [4]. Paraneoplastic syndromes are rare first manifestations of a neoplastic disorder. It can be the first sign of a not yet detected (occult) cancer. Gastroparesis is a manifestation of the paraneoplastic involvement of the neuronal bodies of the gastrointestinal tract. It affects the stomach and is characterised by delayed gastric emptying. Its pathophysiology remains unclear. However, serological detection of anti-Hu autoantibodies suggests an autoimmune destruction of the euronal plexus of the stomach as the main mechanism for its development [5].

Small cell lung cancer represents 15% to 25% of lung cancers. Paraneoplastic syndromes can be early manifestations of this malignancy [6]. In small cell lung cancer neurologic syndromes include the Lambert-Eaton myasthenic syndrome, limbic encephalopathy, polyneuropathy, cerebellar degeneration, retinopathy, opsoclonus-myoclonus, and autonomic neuropathy. Autoimmune mechanisms play a crucial role in their development through autoantibodies that directs against ligand- or voltage-gated channels to cause changes in synaptic function or neuronal excitability [7]. Anti-Hu autoantibodies have been identified to play a crucial role in the pathogenesis of these paraneoplastic neuropathies.

Gastroparesis as first sign of small cell cancer of the lung is very rare. Few case reports have been published [8, 9]. In patients with unexplained gastroparesis, testing for anti-Hu antibodies, as a valuable marker of small cell lung cancer can be considered in the investigation [6, 10].

In one patient presenting with gastroparesis surgery was done and the stomach was removed in order to restore passage of liquids and food. Histological examination of the resected stomach showed absence of ganglion cells with associated chronically inflamed nerve fibres suggestive of gastroparesis due to nerve injury [5].

In general, most studies suggest two complementary approaches for the management of paraneoplastic gastroparesis based on its immune-mediated hypothesis: removal of the antigen source by treating the tumour and suppression of the immune response. The present patient responded well to treatment with steroids and later to chemotherapy. But therapeutic interventions such as diet modifications, pharmacological agents, therapeutic endoscopy, surgery, and psychological counselling are also needed since they contribute to a better outcome [11]. Prokinetic agents (motilin receptor agonists, serotonin 5-HT4 receptor agonists, cisapride, and dopamine antagonists), steroids (dexamethasone), and antiemetics (serotonin 5-HT3 receptor antagonists, tricyclic antidepressants, and phenothiazines) have all been used in the management of paraneoplastic gastroparesis. In the present case this therapy did not have any effect. Once during endoscopy in order to place a new jejunal tube for feeding, some peristalsis was observed. But this remained inefficient.

In retrospect this case exactly reflects another case history reported in the literature. This paper describes a patient with gastroparesis in whom the diagnosis of small cell cancer of the lung ultimately was also made via ultrasound assisted fine needle aspiration. This patient showed high titres of anti-Hu antibodies as well [12].

Paraneoplastic encephalomyelitis is another manifestation of small cell lung cancer. The diagnosis is made via the neurological symptoms and the presence of anti-Hu antibodies in the serum. This manifestation may respond well to treatment with chemotherapy [13].

An old saying is the expression that what you do not know or never have seen you will not recognise easily. This case certainly is a clear example of this statement. It took sometime of diagnostic thinking, a literature search and the consultation of an expert to come to the correct cause of the gastroparesis. The fact that repeated X-rays of the thorax did not show signs of an underlying malignancy certainly made the diagnostic process more difficult.
